# The Role of TEE in Diagnosing Hepatopulmonary Syndrome and Cryptogenic Cirrhosis

**DOI:** 10.1155/2016/9029606

**Published:** 2016-08-31

**Authors:** Joel Scott-Herridge, Kapil Bhagirath, Surinder Janda, Davinder S. Jassal

**Affiliations:** ^1^Department of Internal Medicine, University of Manitoba, Winnipeg, MB, Canada; ^2^Department of Internal Medicine, University of British Columbia, Surrey, BC, Canada; ^3^Section of Cardiology, Department of Internal Medicine, University of Manitoba, Winnipeg, MB, Canada; ^4^Institute of Cardiovascular Sciences, St. Boniface General Hospital, University of Manitoba, Winnipeg, MB, Canada; ^5^Department of Radiology, St. Boniface General Hospital, University of Manitoba, Winnipeg, MB, Canada

## Abstract

In the vast majority of cases, ongoing hypoxemia in a cirrhotic patient is usually hepatopulmonary syndrome (HPS) until proven otherwise; in this case, HPS was suspected prior to any known diagnosis of cirrhosis. This is the first reported case in the literature whereby HPS and cirrhosis were diagnosed after the fact, rather than with the preexisting knowledge of liver cirrhosis.

## 1. Introduction

Hepatopulmonary syndrome (HPS) is considered in individuals with the triad of liver disease, impaired oxygenation, and intrapulmonary microvascular dilatations. The prevalence of HPS among patients with chronic liver disease in the literature is approximately 24% [1]. Although 80% of patients will present with findings of chronic liver disease, the remaining 20% will have new onset dyspnea as their only presenting complaint [2].

Hypoxemia is commonly associated with liver disease, albeit quite nonspecific. A differential diagnosis of hypoxemia in a patient with known chronic liver disease would include hepatic hydrothorax, portopulmonary hypertension, and HPS. In the setting of HPS, severe hypoxemia occurs secondary to shunting through intrapulmonary microvascular dilatations leading to ventilation-perfusion mismatches [3].

We describe a case of a 54-year-old female presenting with unexplained hypoxemia and late transfer of agitated saline contrast from the right-sided circulation into the left atrium on transesophageal echocardiography (TEE), leading to a new diagnosis of HPS due to cryptogenic liver cirrhosis.

## 2. Case Presentation

A 54-year-old Caucasian female with a 15-pack-year smoking history presented for evaluation of hypoxemia. She described NYHA Functional Class III exertional dyspnea over the preceding 7 months, in the absence of any chest tightness or respiratory symptoms of coughing or wheezing. She denied orthopnea, paroxysmal nocturnal dyspnea, or peripheral edema. She did not notice any new rashes, arthralgias, or myalgias to suggest an underlying connective tissue or lung disease. There was no significant travel or exposure history.

On physical examination, her oxygen saturation was 91% on 3 L, and she had blood pressure of 115/76 mm Hg, with a heart rate of 82 beats per minute. On respiratory exam, there were normal breath sounds bilaterally with no wheezes, crackles, or pleural rubs on auscultation. On cardiovascular examination, S1 and S2 were normal, with an early peaking grade II/VI crescendo-decrescendo murmur, loudest at the right upper sternal border radiating to the carotids, consistent with mild to moderate aortic stenosis. There was no evidence of stigmata of chronic liver disease.

Transthoracic echocardiography confirmed preserved left ventricular systolic function with an ejection fraction >60% and mild to moderate subaortic stenosis. There was no evidence of other valvular heart disease and no obvious shunts noted on color Doppler. On TEE, there was evidence of late transfer of agitated saline contrast from the right-sided circulation into the left atrium. During spontaneous respiration, >20 bubbles were identified in the left heart after the 4th beat consistent with a moderate extracardiac shunt (Supplemental Video 1 in Supplementary Material available online at http://dx.doi.org/10.1155/2016/9029606). CT of the chest did not demonstrate any evidence of interstitial lung disease or large pulmonary arteriovenous malformations (AVMs). By diagnosis of exclusion, microvascular intrapulmonary dilatation was suggested as an etiology of the intrapulmonary shunting. An abdominal ultrasound was performed. This raised the possibility of cryptogenic liver cirrhosis, which was confirmed on liver biopsy.

The patient was subsequently assessed by the gastroenterology and hepatology services. There was no evidence of esophageal varices on screening endoscopy. The patient underwent assessment for liver transplantation, but due to multiple comorbidities and concerns about compliance, the patient was deemed not to be a suitable candidate. She is being managed conservatively at present.

## 3. Discussion

Saline contrast echocardiography is a routine technique in the discrimination between intra- and extracardiac shunting. Although an intracardiac shunt is confirmed by the presence of right-to-left shunting across the interatrial septum within 3 cardiac cycles [4], an extracardiac shunt is suggested with late transfer of contrast [5, 6]. The differential diagnosis for a delayed right-to-left shunt on echocardiography is limited and includes pulmonary AVM in the context of Hereditary Hemorrhagic Telangiectasias (HHT), HPS, congenital heart defects with cavopulmonary shunting, and possibly normal variant [7]. As approximately 80% of pulmonary AVMs are associated with HHT [6], CT of the chest is the noninvasive imaging test of choice [6]. In the setting of a normal CT, an abdominal ultrasound should be performed as approximately 10–30% of patients with cirrhosis will have HPS [7].

As a complication of liver disease, HPS is most commonly observed in patients with portal hypertension [8]. Although the pathophysiology of HPS is incompletely understood, it has been hypothesized that the inability of the liver to clear or inhibit nitric oxide, endothelin-1, and tumor necrosis factor-alpha may play a role in the formation of pulmonary AVMs [9]. The clinical manifestations of HPS are the consequences of both hepatic and pulmonary dysfunction; however, clinical findings more specific for HPS include platypnea and orthodeoxia [2]. The diagnosis of HPS requires the triad of liver disease, hypoxemia, and evidence of intrapulmonary shunting on contrast echocardiography [9] and carries with it an increased mortality rate as compared to case matched controls [10]. There are currently no effective medical therapies for the treatment of HPS. Although many approaches to improve gas exchange have been attempted, long term oxygenation and liver transplantation have proven to be the only successful modes of managing patients [8]. For those patients who remain hypoxic after transplantation with large AVMs, intra-arterial coil embolization has been utilized to improve the right-to-left shunt [9].

The present case report is unique and raises some important learning points for the practicing clinician. To begin with, this case illustrates the diagnostic challenge in working up unexplained hypoxemia. After standard workup to rule out respiratory causes of hypoxemia were completed, the patient was sent for a thorough cardiac evaluation. TEE was effective in ruling out an intracardiac shunt, and, moreover, pursuing saline contrast injection demonstrated a moderately significant late bubble transfer. In the absence of clinical features consistent with HHT, the diagnosis of exclusion was HPS. Since the diagnosis of liver cirrhosis was not initially suspected in this patient, the TEE finding led to abdominal ultrasound imaging and liver biopsy to confirm the diagnosis. In the vast majority of cases, ongoing hypoxemia in a cirrhotic patient is usually HPS until proven otherwise; in this case, HPS was suspected prior to any known diagnosis of cirrhosis. This is the first reported case in the literature whereby HPS and cirrhosis were diagnosed after the fact, rather than with the preexisting knowledge of liver cirrhosis.

## 4. Conclusion

The differential diagnosis for a delayed right-to-left shunt on echocardiography is limited and includes HPS. Even in the setting of a negative history for chronic liver disease, HPS may still be the cause for hypoxemia.

## Supplementary Material

Right to left shunting across the inter-atrial septum on transesophageal echocardiography demonstrating late transfer of agitated saline contrast consistent with an extra-cardiac shunt.
